# Advances in Gene Therapy for Rare Diseases: Targeting Functional Haploinsufficiency Through AAV and mRNA Approaches

**DOI:** 10.3390/ijms26020578

**Published:** 2025-01-11

**Authors:** Nuria Bara-Ledesma, Adrian Viteri-Noel, Monica Lopez Rodriguez, Konstantinos Stamatakis, Martin Fabregate, Almudena Vazquez-Santos, Vicente Gomez del Olmo

**Affiliations:** 1 Internal Medicine Department, Hospital Universitario Ramón y Cajal, IRYCIS, 28034 Madrid, Spain; nuria.bara@salud.madrid.org (N.B.-L.);; 2 Faculty of Medicine and Health Sciences, Universidad de Alcalá (UAH), 28805 Alcalá de Henares, Spain; 3 Department of Molecular Biology, Universidad Autónoma de Madrid, IRYCIS, 28049 Madrid, Spain

**Keywords:** rare diseases, gene therapy, adeno-associated virus (AAV), messenger RNA (mRNA), functional haploinsufficiency, transgene expression

## Abstract

Most rare diseases (RDs) encompass a diverse group of inherited disorders that affect millions of people worldwide. A significant proportion of these diseases are driven by functional haploinsufficiency, which is caused by pathogenic genetic variants. Currently, most treatments for RDs are limited to symptom management, emphasizing the need for therapies that directly address genetic deficiencies. Recent advancements in gene therapy, particularly with adeno-associated viruses (AAVs) and lipid nanoparticle-encapsulated messenger RNA (mRNA), have introduced promising therapeutic approaches. AAV vectors offer durable gene expression, extensive tissue tropism, and a safety profile that makes them a leading choice for gene delivery; however, limitations remain, including packaging size and immune response. In contrast, mRNA therapeutics, formulated in LNPs, facilitate transient protein expression without the risk of genomic integration, supporting repeated dosing and pharmacokinetic control, though with less long-term expression than AAVs. This review analyzes the latest developments in AAV and mRNA technologies for rare monogenic disorders, focusing on preclinical and clinical outcomes, vector design, and delivery challenges. We also address key regulatory and immunological considerations impacting therapeutic success. Together, these advancements in AAV and mRNA technology underscore a new era in RD treatment, providing innovative tools to target the genetic root of these diseases and expanding therapeutic approaches for patients who currently face limited medical options.

## 1. Introduction

Rare diseases (RDs) comprise a heterogeneous group of over 7000 distinct disorders that, collectively, affect a considerable portion of the population. According to the European Commission, up to 36 million individuals within the EU live with an RD, representing a significant healthcare challenge [1]. Around 72% of RD have a genetic origin, and 70% have their onset during childhood. Without treatments targeting the underlying etiology, management of RD symptoms is often inefficient and ineffective, resulting in worsened patient outcomes and increased healthcare resource consumption. Notably, while RDs affect only 5% to 7% of the population, they account for nearly 10% of total direct healthcare expenditures in developed countries [1,2].

Given the genetic origin of most RDs, these conditions often arise from a wide spectrum of pathogenic genetic variants. Functional haploinsufficiency, a condition where one functional gene copy is unable to maintain normal physiological function, emerges as a key mechanism underlying many RDs. This phenomenon often stems from mutations, deletions, or disruptions in one allele, causing reduced gene expression or protein function (Table 1). Therapeutic approaches targeting haploinsufficiency aim to restore protein levels to functional thresholds by increasing gene expression, stabilizing protein products, or reducing degradation [3].

Traditional treatments for RDs, such as small-molecule drugs, generally cannot fully compensate for missing or dysfunctional genes. Biologic therapies, including protein replacement, offer partial relief but are limited by their inability to restore normal cellular homeostasis. In recent years, gene therapy has emerged as a promising strategy to directly address the underlying genetic causes of RDs. Approaches in gene therapy include (1) replacing defective genes with functional copies, (2) silencing pathogenic genes, and (3) introducing or overexpressing therapeutic genes or synthetic constructs [9]. These interventions can be delivered in vivo, through direct patient administration, or ex vivo, where cells are modified outside the body and subsequently reintroduced.

Among the various vector systems, adeno-associated viruses (AAVs) have gained prominence due to their capacity for targeted and transient gene expression without genomic integration. However, challenges persist, including limited vector packaging capacity, potential immune responses, and the need for improved delivery efficiency [10,11]. In addition to viral vectors, messenger RNA (mRNA)-based therapies have emerged as a promising alternative, particularly following the demonstrated success of mRNA vaccines during the COVID-19 pandemic. Encapsulated within lipid nanoparticles (LNPs), mRNA can be delivered safely to target cells, enabling transient protein expression without the risk of genomic integration. This platform offers distinct advantages for RDs that require controlled and repeatable dosing, while also mitigating many of the immunogenic challenges associated with viral vectors (Table 2). Together, gene- and mRNA-based therapies represent innovative strategies for addressing RDs driven by functional haploinsufficiency, providing new possibilities for treating conditions that have historically lacked effective therapeutic options [12].

## 2. Adeno-Associated Virus

Since the early 2000s, AAV has become a widely explored vector for gene therapy due to its safety profile and effectiveness in in vivo gene delivery. AAV is a small (25 nm), non-enveloped virus from the *Parvoviridae* family, with an icosahedral capsid that accommodates a single-stranded DNA genome of approximately 4.7 kb [9]. The viral genome includes essential non-structural proteins (Rep), capsid proteins (VP1–3), and an assembly-activating protein (AAP), all flanked by inverted terminal repeats (ITRs), which are crucial for replication and packaging [13]. Recombinant AAVs (rAAVs) are engineered to retain only the ITRs, replacing the rest of the genome with a therapeutic expression cassette. This cassette typically includes the gene of interest, along with a promoter and regulatory elements, making rAAVs particularly well suited for therapeutic applications.

**Figure 1:**
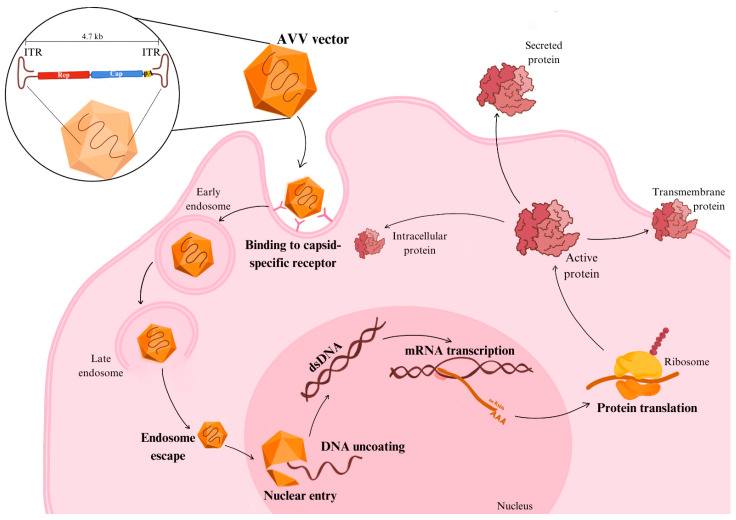
Mechanism of action of AAV vectors in gene therapy. AAVs bind to specific cell surface receptors and are internalized via endocytosis. After escaping the endosomes, the AAVs are trafficked to the nucleus and the capsid is uncoated. The single-stranded DNA genome is converted into double-stranded DNA. The transgene is then transcribed into mRNA, exported to the cytoplasm, and translated into therapeutic protein. AAVs: adeno-associated viruses; DNA: deoxyribonucleic acid; ITR inverted terminal repeat.

AAVs enter cells via receptor-mediated endocytosis and traffic through the endosomal pathway. Following endosomal escape, the viral particles reach the nucleus, where their single-stranded genome is converted into double-stranded DNA by host cell polymerases (Figure 1) [14]. Notably, rAAVs generally persist as episomes within the nucleus, avoiding integration into the host genome. This feature minimizes genotoxic risks and supports their established reputation as safe vectors for long-term transgene expression [15,16]. Nonetheless, integration events, though infrequent (0.1–1% of cases), have prompted ongoing research into their long-term safety profile [17,18].

AAV vectors present several advantages as gene delivery tools. They exhibit broad tissue tropism, low immunogenicity compared with other viral vectors, and sustained gene expression, making them suitable candidates for gene therapy [19]. However, their limited packaging capacity (~5.0 kb) poses a challenge for delivering large therapeutic genes [20,21]. Solutions include engineering truncated yet functional forms of larger genes to fit within the vector, although this approach is gene-specific and technically complex [14].

Although the immune response to AAVs is lower than that to other engineered viruses, it remains a significant clinical hurdle. As described in several gene therapy trials, the capsid proteins can trigger innate and adaptive immunity, resulting in inflammation and potential complement activation shortly after administration [22]. Additionally, pre-existing neutralizing antibodies (NAbs) against AAV, observed in 20–80% of the population depending on the serotype, can substantially reduce therapeutic efficacy, particularly in cases of systemic administration [17,23]. This response limits clinical translation, as many trials must exclude patients with detectable NAbs [24]. Notably, the prevalence of seropositivity increases with age, posing a particular challenge for clinical applications given that most RDs are chronic and tend to progress over time [9]. Cross-reactive NAbs, formed due to structural similarities among AAV serotypes, further limit re-dosing and affect subsequent treatments with other AAV variants [25]. The levels of NAbs are influenced by a range of patient- and therapy-related factors, including patient demographics, disease status, capsid type, transgene characteristics, and dosage [19]. However, it has been suggested that some AAV serotypes may be less sensitive to antibody neutralization, as observed in a recent trial of AAV5 gene therapy against hemophilia B [26].

Strategies to circumvent immunogenicity include capsid modifications, reducing vector doses, tissue-specific targeting, and immunosuppressive regimens [27]. Additionally, embedding miRNA binding sites (miR-BSs) within rAAV vectors has shown promise in suppressing transgene expression in antigen-presenting cells, thereby reducing immune responses and enhancing transgene persistence. Hence, incorporating miR-142 binding sites into rAAV1 vectors, for example, has been shown to effectively repress costimulatory signals in dendritic cells, mitigate the cytotoxic T-cell response, and reduce the clearance of transduced muscle cells in murine models. Likewise, this approach has been demonstrated to enable sustained transgene expression in myofibers with minimal production of anti-transgene IgG [28].

In practical terms, rAAVs maintain the capsid structure of wild-type AAV but replace all viral protein-coding sequences with the therapeutic cassette. The inclusion of only ITRs, essential for genome replication and vector production, maximizes the usable packaging space and reduces cytotoxicity. Standard molecular cloning techniques enable precise modifications of the rAAV genome, making them a versatile tool in gene therapy. Despite their payload limitations, rAAV vectors provide a stable platform for expressing genes in diverse tissues, offering significant potential for advancing therapeutic approaches in RDs [14].

### 2.1. Preclinical Studies

Extensive proof-of-concept studies have shown the potential of AAV-mediated gene therapy in various animal models that emulate the human phenotypes of several inborn metabolic disorders. Promising outcomes have been reported in conditions such as phenylketonuria, Gaucher disease, urea cycle disorders, glycogen storage disease type Ia, organic acidurias, lipoprotein lipase deficiency, Pompe disease, and mucopolysaccharidosis [24].

For lysosomal storage diseases, certain AAV serotypes, specifically AAV9, AAV8, and AAVrh.10, have shown efficacy as effective delivery vectors [29]. Additionally, two engineered capsids, AAVS3 and 4D-C102, have emerged as promising variants with enhanced specificity and transduction efficiency. AAVS3 has been optimized for human liver tropism through strategic sequence modifications, achieving high hepatocyte transduction rates, which may be particularly beneficial for liver-targeted diseases [30]. Meanwhile, 4D-C102, a muscle-specific variant developed through directed evolution, exhibits superior gene delivery capabilities and reduced immunogenicity in cardiac and hepatic cells compared to commonly used serotypes AAV1, AAV8, and AAV9. This variant is currently under clinical evaluation for the treatment of Fabry disease (NCT04519749) [9].

In the context of muscular disorders, Potter, R.A. et al. [31] evaluated the safety and efficacy of systemically administered, dose-escalated AAV–micro-dystrophin in the mdx mouse model of Duchenne muscular dystrophy. The results demonstrated dose-dependent improvements in muscle function and dystrophin expression, with the highest doses achieving near-normal muscle morphology and significantly reduced fibrosis. These findings highlight the potential of AAV vectors to restore function in monogenic muscular disorders.

Nevertheless, despite the encouraging results of preclinical studies, several translational challenges remain to be addressed. Notably, the limited packaging capacity of AAV vectors restricts their use for larger genes, and the potential for immune responses to the viral capsid and transgene poses additional limitations. Therefore, advancing these therapies from preclinical models to clinical applications will require continued efforts in capsid engineering, immune modulation strategies, and optimization of dosing regimens [9].

### 2.2. Clinical Trials

In recent years, AAV has emerged as a key vector in gene therapy, with its structural simplicity, favorable safety profile, and molecular versatility propelling over 100 active clinical trials by 2024 [32]. The first AAV-based gene therapy approved in humans was Glybera for lipoprotein lipase deficiency, authorized by the European Medicines Agency (EMA) in 2012 [33]. Currently, six AAV-based therapies have received approval from U.S. Food and Drug Administration, while nine are EMA-approved, targeting conditions such as Duchenne muscular dystrophy, hemophilia B, hemophilia A, metachromatic leukodystrophy, retinal dystrophy, and spinal muscular atrophy. Many of these therapies are undergoing post-marketing observational studies to further evaluate their long-term safety and efficacy.

Approximately 60 ongoing AAV-based trials are currently in early-phase stages (Phase I and II), primarily targeting RDs such as hemophilia, degenerative neurological conditions, retinopathies, metabolic disorders, and lysosomal diseases. However, most AAV trials remain in early development, with nearly 75% still in Phase I or Phase I/II stages and limited advancement to later stages. To date, around 40 Phase I and II studies have been completed and registered on ClinicalTrials.gov. In contrast, only five active Phase III trials are currently registered, underscoring the challenges of transitioning these therapies into late-stage clinical development. Table 3 provides an overview of the six FDA-approved AAV-based therapies that have reached the market.

There is a wide spectrum of diseases with diverse manifestations that stand to benefit from similar treatment technology. For example, significant progress has been made in tailoring AAV capsids to specific tissues and disease profiles. Among the 17 AAV capsid types and 27 promoters identified in clinical trials, AAV2 is the most frequently used, especially for ocular and central nervous system disorders. Other capsids demonstrate tissue-specific preferences: AAV8 for blood disorders, AAV9 for lysosomal diseases, and AAV1 and AAV9 for neuromuscular disorders. Thus, the SPRINT and STR1VE trials of onasemnogene abeparvovec in spinal muscular atrophy (SMA) patients confirmed the efficacy of a single intravenous dose, achieving substantial motor function improvements in presymptomatic infants [34]. Notably, the STR1VE study evaluated the same therapy in SMA type 1, showing its efficacy and safety with the administration of a single intravenous dose in symptomatic patients under six months of age [35]. The sole adverse effects observed were elevated transaminases, which were effectively managed with steroid-based therapies, and a transient thrombocytopenia. Likewise, onasemnogene abeparvovec was effective and well tolerated in presymptomatic infants at risk for SMA type 2. This gene therapy product is authorized and currently commercially available in Europe.

Other applications include Valoctocogene Roxaparvovec, an AAV5 vector delivering factor VIII for hemophilia A, which demonstrated sustained factor levels and reduced bleeding at one year [36]. A total of 134 participants, aged 18 years and above, with no prior history of anti-AAV5 antibodies, were enrolled in the Phase III clinical trial. Each subject received a single intravenous dose of the product. At four weeks of treatment, patients exhibited reduced bleeding, while at week 52, they still displayed elevated levels of Factor VIII, compared to those receiving Factor VIII alone. Similarly, gene therapy for hemophilia B has shown safety and effectiveness in reducing bleeding when factor IX is delivered through AAV5. These therapies are now commercially available [36]. In the case of aromatic L-amino acid decarboxylase deficiency, Phase I and II clinical trials use an infusion of the affected gene via an AAV administered directly [37]. The Phase II study evaluated seven children aged 4–9 years old, showing motor improvements during a follow-up period of 18 months.

Acute intermittent porphyria is caused by haploinsufficiency of porphobilinogen deaminase, resulting in hepatic overproduction of neuro-toxic heme precursors, namely porphobilinogen and delta-aminolevulinic acid (ALA), which trigger neurovisceral attacks. A Phase I, open-label, dose-escalation, multicenter clinical trial investigated the safety of an AAV5-based gene therapy for the treatment of acute intermittent porphyria [38]. Four cohorts of two patients each received a single intravenous injection at doses ranging from 5 × 10^11^ to 1.8 × 10^13^ genome copies/kg, with no serious adverse events attributed to AAV5 administration. As expected, all patients developed neutralizing antibodies against AAV5, yet importantly, no cellular immune response was observed.

Pompe disease is an inherited neuromuscular disorder caused by a deficiency in the lysosomal enzyme acid alpha-glucosidase (GAA). Current Phase I/II clinical trials aim to improve respiratory function in pediatric patients with Pompe disease who rely on mechanical ventilation. Although safety has been well evaluated, the small sample sizes have so far limited the conclusiveness of efficacy data. Among ongoing investigations, one trial employs an adenoviral vector, while another uses a recombinant AAV vector associated with a cytomegalovirus promoter. Additionally, a Phase I study is evaluating the potential safety benefits of administering rituximab seven days prior to AAV gene therapy to mitigate immune-related challenges [39].

In parallel, research on lysosomal storage disorders continues to advance. For Fabry disease, early-phase clinical trials have completed participant recruitment, with results pending publication [40]. Fabry disease (FD), an X-linked metabolic disorder, results from pathogenic variants in the *GLA* gene that cause functional haploinsufficiency of the lysosomal enzyme alpha-galactosidase A, leading to accumulation of glycosphingolipids like Gb3 and lyso-Gb3 throughout the body. Additionally, an active Phase I clinical trial involves a single administration of a recombinant adenovirus vector encoding α-Gal A cDNA, with results also pending (NCT06207552). Similarly, there are three ongoing Phase I and II trials for Gaucher disease. Notably, trial NCT06272149 employs AAV serotype 9 to deliver the human GBA1 gene. As with Fabry disease, most outcomes for these trials remain unpublished at this stage. Table 4 summarizes several clinical trials of AAV-based therapies, including their targeted diseases, therapeutic product, trial phase, and preliminary results.

Despite these promising advances, immune-related challenges remain a barrier. Approximately 45% of AAV-based clinical trials exclude patients with pre-existing NAbs against AAV capsids to prevent immune-mediated toxicities, with exclusion rates reaching nearly 90% in studies targeting for blood disorders [41]. Although immunosuppressive regimens, primarily corticosteroids and other immunomodulatory agents, are commonly employed, their use is inconsistently reported across trials. Developing effective strategies to mitigate the immune response to AAV, such as capsid modifications and miRNA-mediated immunomodulation, is crucial for enhancing patient eligibility and therapeutic outcomes. Many drugs, such as rapamycin, an mTOR inhibitor, are being studied for their ability to selectively suppress the activation of naive adaptive immune cells while preserving recall immune responses. Similarly, reducing inflammatory signals by modifying pathogen-associated molecular patterns (PAMPs) on AAV vectors may mitigate primary immune responses but is unlikely to significantly impact the reactivation of memory immune cells [42]. The immune response, along with other factors such as vector tropism and intracellular barriers, limits the efficiency of AAV-mediated gene transfer. To overcome these challenges, higher vector doses are often required to achieve therapeutic efficacy, which increases the risk of systemic toxicity, including acute liver injury, atypical hemolytic uremic syndrome, and thrombotic microangiopathy [43,44].

While AAV-mediated gene therapy is still maturing, its rapid progress has paved the way for broader application in treating rare genetic diseases. The growing interest of the scientific community in AAV for gene therapy is evidenced by a substantial rise in publications: from 1619 articles indexed in PubMed between 2005 and 2015 to 3681 articles from 2015 to 2024. For RDs with limited treatment options, AAV-based gene therapy represents a promising avenue, bolstered by fast-track and orphan drug designations that encourage further research [9]. Although additional optimization and comprehensive safety evaluation are required, current advancements underscore the potential of AAV-based approaches to improve patient outcomes and quality of life.

## 3. Messenger RNA (mRNA)

**Figure 2:**
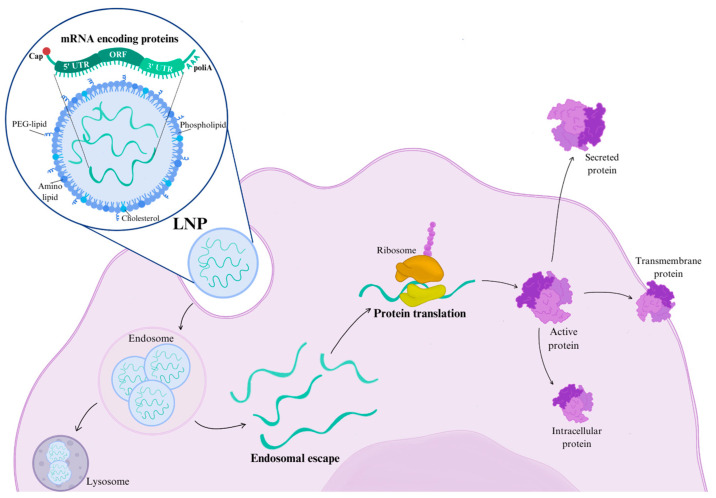
Mechanism of action of LNP-encapsulated mRNA in gene therapy. LNPs deliver therapeutic mRNA into target cells, where the mRNA is released from endosomes into the cytoplasm. Once in the cytoplasm, the mRNA is translated into the corresponding protein, which then reaches its intracellular or extracellular locations. LNP: lipid nanoparticles; mRNA: messenger ribonucleic acid; PEG-lipid: polyethylene glycol-lipid.

The chronic nature of monogenic disorders often necessitates repeated treatments, posing significant challenges for viral vector use due to immune responses that hinder re-administration. In this context, mRNA therapy has emerged as a promising alternative, offering the flexibility to encode a wide range of proteins, including transmembrane, mitochondrial, intracellular, and secreted proteins [45]. Therapeutic mRNA is engineered with (i) a human codon-optimized protein-coding sequence, (ii) 5′ and 3′ untranslated regions (UTRs) to enhance translation and stability, (iii) a 7-methyl guanosine cap to initiate translation, and (iv) a polyadenosine tail (100–120 bases) to further stabilize the mRNA molecule (Figure 2) [46].

Exogenous mRNA is highly susceptible to degradation by extracellular ribonucleases, making a robust delivery vehicle essential to ensure therapeutic efficacy. The recent success of mRNA vaccines for SARS-CoV-2, including mRNA-1273 (Moderna) and BNT162b2 (Pfizer/BioNTech), has demonstrated the safety and effectiveness of lipid nanoparticles (LNPs) for non-viral delivery. These vaccines encapsulate mRNA within LNPs, which are composed of ionizable lipids, sterols, phospholipids, and polyethylene glycol (PEG)-lipids. This structure provides a protective barrier that shields the mRNA from RNase-mediated degradation, thereby enhancing its stability and bioavailability [45].

LNP-encapsulated mRNA requires only cytoplasmic delivery to initiate protein translation, bypassing the need for nuclear entry and reducing the risk of genomic integration. This approach offers additional advantages over AAV, including the ability to provide controlled and transient protein expression tailored to specific pharmacokinetic and dosing requirements. LNPs facilitate mRNA internalization through opsonization by apolipoprotein E (ApoE) and subsequent low-density lipoprotein (LDL) receptor-mediated endocytosis, followed by endosomal escape into the cytosol for translation into functional protein. Recent advancements in LNP technology have further improved endosomal escape efficiency, biodegradability, and safety profiles, as demonstrated in animal studies, including non-human primate models.

### 3.1. Preclinical Studies

Preclinical studies have explored mRNA-based therapies for a range of RDs, focusing particularly on conditions where enzyme deficiency is primarily or exclusively hepatic. The liver represents a key target in these studies, as mRNA LNPs are naturally coated by apolipoproteins in the bloodstream, facilitating their uptake by hepatocytes via LDL receptors [47].

Zhu et al. [48] provided proof-of-concept evidence for mRNA encoding the α-Gal A enzyme in a Fabry disease mouse model and wild-type non-human primates. In a Fabry disease mouse model, a single intravenous bolus of LNP-encapsulated α-Gal A mRNA (0.05–0.5 mg/kg) restored α-Gal A enzyme levels in key organs (liver, spleen, heart, and kidney) and significantly reduced glycosphingolipid accumulation. The effects persisted, with tissue levels of Gb3 and lyso-Gb3 remaining low for 5–6 weeks after a single dose and showing no significant rebound until 12 weeks. Importantly, no anti-α-Gal A antibodies were detected after repeated dosing, unlike traditional enzyme replacement therapy (ERT), where immune responses are often triggered within 2–3 doses. These findings suggest that mRNA therapy may offer improved immune tolerance compared to ERT by facilitating endogenous post-translational modifications in the liver.

mRNA technology has also been explored for treating mitochondrial enzyme deficiency disorders, such as propionic acidemia, a rare metabolic condition caused by propionyl-CoA-carboxylase (PCC) deficiency. In a mouse model of propionic acidemia, single intravenous administration of mRNA-3927 (1 mg/kg) achieved physiologically relevant levels of functional enzyme, detectable for up to 21 days [49]. Subsequent repeated dosing administered either tri-weekly or monthly over 3–6 months led to normalized key biomarkers, including 2MC, ammonia, 3HP, and C3. Notably, liver toxicity markers remained within normal ranges, with only mild immune cell infiltration observed around the hepatic vein. These promising preclinical results have paved the way for ongoing Phase I/II clinical trials for propionic acidemia (NCT04159103, NCT05130437).

Moreover, preclinical studies have also shown favorable results in other RDs, including glycogen storage disorder type Ia [50,51], acute intermittent porphyria [52], cystic fibrosis, and primary ciliary dyskinesia. Importantly, for primary ciliary dyskinesia, aerosol-mediated mRNA delivery in rats and non-human primates was shown to be non-toxic [45], demonstrating the potential of mRNA as a therapeutic option for respiratory diseases. To enhance the targeting of mRNA therapies to various tissues, both passive and active strategies are being investigated. Passive targeting involves modifying the lipid composition of LNPs to achieve organ specificity, while active targeting uses small-molecule ligands, peptides, or tissue-targeting synthetic peptides to direct LNPs to specific cell types [53]. Current research is particularly focused on overcoming the challenges of targeting tissues, such as the brain, muscles, and neurons, using peptide-mediated delivery methods.

### 3.2. Clinical Trials

As previously discussed, LNPs represent a novel method for drug and genetic material delivery, offering an alternative to AAVs. The use of LNPs for mRNA delivery gained momentum following the COVID-19 pandemic, during which LNP-encapsulated mRNA vaccines became the first to receive approval from U.S. and European regulatory agencies. This rapid adoption has led to a majority of mRNA-related clinical trials focusing on infectious diseases. However, the potential of mRNA therapies in treating monogenic disorders is increasingly being explored, with initial Phase I and II trials beginning as early as 2020 [45].

Currently, seven clinical trials are investigating mRNA-based therapies for rare genetic disorders. These trials target a range of conditions, including glycogen storage disease type 1a (NCT05095727), methylmalonic acidemia (NCT04899310), propionic acidemia (NCT04159103 and NCT05130437), cystic fibrosis (NCT05668741 and NCT03375047), ornithine transcarbamylase deficiency (NCT04442347), and primary ciliary dyskinesia (NCT05737485). Notably, propionic acidemia is the only rare disease for which systemic mRNA therapy has progressed beyond the dose-optimization stage of Phase I/II trials. Preliminary data have suggested both safety and potential clinical benefits at various dosing levels, and the trial has now entered the dose-expansion phase to establish optimal therapeutic dosing (NCT05130437 2021a). Table 5 summarizes the clinical trials for mRNA-based therapies in 2024, highlighting their targeted diseases, therapeutic product, trial phase, and preliminary outcomes, providing a comprehensive overview of ongoing advancements in the field.

Most lipid-based mRNA therapeutics in ongoing clinical trials are administered intravenously, despite their wide range of potential applications. Intravenous delivery is particularly advantageous for immunotherapeutic applications, as it allows high levels of antigen production compared to other administration routes [54]. However, systemic delivery requires larger drug volumes and higher doses compared to tissue-specific approaches, resulting in drug accumulation in the liver, which can limit its availability in target tissues [55]. This accumulation may also lead to side effects, such as hepatic inflammation or necrosis [56,57]. Inhalation has emerged as an alternative method for targeted administration, with potential applications in both therapeutics and vaccines. Advances in nebulization and lipid nanoparticle formulation have increased interest in this approach [58]. Currently, the inhalable mRNA therapeutics ARCT-032 and VX-522, both designed as protein replacement therapies for cystic fibrosis, are the only such products in clinical trials [59].

To address the delivery challenges of mRNA-based therapies, particularly to non-hepatic targets, various strategies are being explored to enhance tissue specificity and transfection efficiency. Advances in lipid nanoparticle (LNP) formulations remain at the forefront, with modifications to ionizable lipids and the incorporation of targeting ligands showing promise for selective delivery [60]. Passive targeting approaches optimize the physicochemical properties of LNPs, such as size, charge, and lipid composition, to exploit natural biodistribution patterns [53]. Active targeting strategies involve functionalizing LNPs with small-molecule ligands, antibodies, or synthetic peptides that bind to specific receptors expressed on target tissues, such as integrins or endothelial markers. Another approach is based on peptides which can facilitate tissue penetration and endosomal escape, critical for efficient mRNA delivery and expression [61]. Moreover, engineered polymers and hybrid nanoparticle systems combining lipid and polymeric materials are being developed to improve stability and enhance delivery efficiency [60]. These innovations aim to overcome the inherent liver tropism of LNPs, broadening the therapeutic scope of mRNA technologies and enabling their application to a wider range of diseases, including neurological, muscular, and respiratory conditions.

These initial trials highlight the promising potential of mRNA therapeutics for addressing rare genetic disorders. Although still in early stages, the results suggest favorable safety, tolerability, and efficacy profiles across various monogenic conditions. As clinical research continues to refine dosing strategies and optimize LNP delivery systems, mRNA-based therapies could provide a flexible, targeted alternative to traditional gene therapies. Further advancements in LNP technology are expected to enhance delivery efficiency and stability, opening the door to personalized mRNA therapies tailored to the specific needs of patients with rare genetic diseases.

## 4. Conclusions

The development of both viral and non-viral vectors, such as AAVs and LNPs, has provided promising platforms that enable targeted and efficient delivery of genetic material. While viral vectors continue to offer long-term transgene expression, mRNA technologies, particularly when encapsulated in LNPs, present alternatives that avoid genomic integration and are well suited for repeated dosing. Despite remaining challenges, including immunogenicity, vector capacity limitations, and the need for optimized dosing strategies, ongoing preclinical and clinical studies demonstrate significant potential for these therapies to safely and effectively address the underlying genetic causes of RDs. Certain issues such as immune or allergic reactions must be continuously evaluated for both approaches. Furthermore, considerations regarding local/tissue-directed versus systemic administration, depending on the disease characteristics, should be incorporated into all studies. With continued innovation and optimization, these novel approaches hold the promise of transforming patient care and expanding therapeutic options for populations that have historically faced limited medical interventions.

## Figures and Tables

**Table 1:** Examples of hereditary diseases caused by gene haploinsufficiency.

Disease Name	Gene	Incidence/100,000	Reference
Hereditary hemorrhagic telangiectasia	*ENG/ACVRL1/* *SMAD4*	10–20	[4]
Marfan syndrome	*FBN1*	2–3	[5]
Microphthalmia with linear skin defects syndrome	*HCCS*	0.1	[6]
Familial adenomatous polyposis	*APC*	1	[7]
Ehlers–Danlos syndrome	*COL3A1/COL5A1/* *COL5A2*	1–2	[8]

**Table 2:** Comparison of key differences between AAV- and mRNA-based therapies in gene therapy for rare diseases.

	Adeno-Associated Virus (AAV)	Messenger RNA (mRNA)
Mechanism of action	Delivers genetic material directly to the nucleus for long-term gene expression	Remains in the cytoplasm; enables transient protein expression
Size of transgene	4.7 kb	No limit
integration	Low but possible, depending on serotype and vector design	No risk of genomic integration
Dosing flexibility	Single administration often sufficient for long-term effect	Requires repeated dosing due to transient expression
Immunogenicity	Potential for immune response to viral capsid; pre-existing immunity may limit efficacy	Lower immunogenicity; mRNA delivery systems (e.g., LNPs) can still trigger immune response

**Table 3:** FDA-approved AAV-based gene therapy products.

Product	Company	Indication	AAV Serotype	Approval Year
LUXTURNA (voretigene nepar-vovec-rzyl)	Spark Therapeutics, Inc. Philadelphia, PA, United States.	Biallelic RPE65 mutation-associated retinal dystrophy	AAV2	2017
ZOLGENSMA (onasemnogene abepar-vovec-xioi)	Novartis Gene Therapies, Inc. Zurich, Switzerland.	Spinal muscular atrophy (SMA)	AAV9	2019
HEMGENIX (etranacogene dezapar-vovec-drlb)	CSL Behring LLC. King of Prussia, PA, United States.	Hemophilia B	AAV5	2022
ROCTAVIAN (valoctocogene roxapar-vovec-rvox)	BioMarin Pharmaceutical Inc. San Rafael, CA, United States.	Hemophilia A	AAV5	2023
ELEVIDYS (delandistro-gene moxepar-vovec-rokl)	Sarepta Therapeutics, Inc. Cambridge, MA, United States.	Duchenne muscular dystrophy (DMD)	AAVrh74	2023
BEQVEZ (fi-danacogene elapar-vovec-dzkt)	Pfizer, Inc. New York, NY, United States.	Hemophilia B	AAVRh74	2024

**Table 4:** Summary of key clinical trials for AAV-based therapies.

Disease	Product	Phase	Outcomes	Identifier
Spinal Muscular Atrophy	Onasemnogene Abeparvovec-xioi (AAV9)	III	Completed; significant motor function improvements; three serious adverse events were related or possibly related to the treatment (two patients had elevated hepatic aminotransferases, and one had hydrocephalus).	NCT03306277
Hemophilia A	Valoctocogene Roxaparvovec (AAV5)	III	Active; interim data show sustained Factor VIII levels and reduced bleeding episodes at 52 weeks.	NCT03370913
Hemophilia B	FLT180a (AAVS3)	I/II	Terminated early due to challenges during the COVID-19 pandemic and a change in requirements of data to be submitted for marketing authorization; initial results showed a reduction in bleeding episodes with sustained Factor IX levels.	NCT03369444
Pompe Disease	AT845 (AAV8)	I/II	Safety evaluation ongoing; preliminary data suggest good tolerability.	NCT04174105
Mucopolysaccharidosis Type IIIA	ABO-102 (AAV9)	II/III	Safety and efficacy studies ongoing; improvements in cognitive scores in early results.	NCT02716246
Duchenne Muscular Dystrophy	Delandistrogene moxeparvovec	I/II	Completed; well tolerated with minimal adverse events; robust micro-dystrophin expression and localization; improved creatine kinase levels.	NCT03375164
Pompe Disease	Zocaglusagene nuzaparvovec (AAV8)	I/II	Active; safety established; efficacy data pending.	NCT04174105

**Table 5:** Summary of key clinical trials for mRNA-based therapies.

Disease	Product	Phase	Outcomes	Identifier
Glycogen Storage Disease Type 1a	mRNA-3745	Ia	Ongoing; evaluating safety and efficacy in metabolic normalization.	NCT05095727
Methylmalonic Acidemia	mRNA-3705	I/II	Active; studying dose-dependent biomarker normalization.	NCT04899310
Propionic Acidemia	mRNA-3927	I/II	Dose-escalation studies ongoing; normalization of metabolic biomarkers reported.	NCT04159103, NCT05130437
Cystic Fibrosis	VX-522 (CFTR-mRNA, aerosol delivery)	I/II	Recruitment ongoing; preclinical safety data demonstrated.	NCT05668741, NCT03375047
Ornithine Transcarbamylase Deficiency	ARCT-810 (Ornithine Transcarbamylase mRNA)	Ib	Completed; pending publication of safety and efficacy results.	NCT04442347
Primary Ciliary Dyskinesia	RCT1100 (targeted mRNA)	I	Active; safety and dose-escalation studies in progress.	NCT05737485

CFTR: cystic fibrosis transmembrane conductance regulator.
